# Addressing human rights abuses at mega-sporting events—A shared responsibility in theory and practice

**DOI:** 10.3389/fspor.2022.1067088

**Published:** 2023-02-23

**Authors:** Daniela Heerdt

**Affiliations:** ^1^ T.M.C. Asser Institute, The Hague, Netherlands; ^2^ Independent Consultant on Sport and Human Rights, Utrecht, Netherlands

**Keywords:** mega-sporting events, human rights abuses, shared responsibility, collaborative remedy, research, education

## Abstract

Mega-sporting events (MSEs) have great potential to promote human rights and be a force for good, not only in the host country or city where the event takes place, but also beyond. At the same time, these events are regularly linked to human rights abuses and this opinion piece provides an overview of MSE-related adverse human rights impacts and discusses how the governance of these events enables these impacts to occur in the context of bidding for, preparing, and delivering these events. At the core of this discussion the paper presents a shared responsibility approach and argues that if applied in a preventative and retrospective way and including the concept of collaborative remedy, it can help address these adverse impacts. This is followed by a reflection on the feasibility of this approach, by considering to what extent current developments in the evolving sport and human rights movement are going in that direction and the paper finishes with a brief discussion on the importance of making the current changes sustainable and how research and education are key elements of this endeavour.

## Introduction: human rights impacts of MSEs

MSEs like the Olympic and Paralympic Games (OPGs), the FIFA World Cup, the Commonwealth Games, UEFA's EURO Championship and many others, are complex events from a number of perspectives, in particular when considering their human rights impacts. On the one hand, they have the potential to promote human rights and be a force for good, not only in the host country or city where the event takes place, but also beyond. Interestingly, and based on Qatar's World Cup story, it has recently even been argued that it is possible to speak of a human rights legacy of these events in positive terms (1). However, and on the other hand, it is a fact that these events are often linked to human rights abuses, in particular cases of forced evictions without due process or compensation, abuse and exploitation of workers and migrant workers, silencing of civil society and human rights defenders, harassment and arrests of journalists, or other types of discrimination against those participating in the event as athletes, fans, entourage, or volunteers, but also against local communities (2). This has been documented extensively in the past decades by civil society and increasingly also academia (3).

These abuses can arguably be traced back to two different kinds of origins. First, they happen as a result of actions and decisions taken in the context of organizing and delivering the event. If due to the hosting of the event, decisions are being made to move neighbourhoods and relocate people, and this is not done with respect of their rights to information, participation, and compensation, then that would be an example for actions and decisions taken for a specific MSE, that resulted in human rights abuses. In fact, forced evictions took place ahead of many of the past MSEs. A study by the Centre on Housing Rights and Evictions shows that for the Seoul, Barcelona, Atlanta, Sydney, Athens, Beijing, and London events in total around 2 million families and individuals have been displaced or forcefully evicted due to construction projects (4). In the course of the organization of the Beijing Olympics alone almost 1.5 million people were evicted (5). Displacements do not automatically lead to human rights abuses, but are illegal if no adequate compensation is secured for the loss of property and even more so if they are carried out forcefully, which unfortunately is often the case if they happen in the context of organizing an MSE (6).

Other examples of MSE-related human rights abuses that are linked to decisions or actions taken for the event can include human rights abuses that occur in the supply chain of a certain event, in relation to procurement of goods that are needed for infrastructure, but also merchandise or apparel (7). Additional human rights risks caused by measures adopted for the delivery of an MSE emanate from increased safety and security measures, such as the introduction of surveillance software. For the World Cup 2018, Russia installed thousands of surveillance cameras, including facial recognition software, which can infringe rights to privacy (8).

The second origin of these abuses is arguably related to the fact that the organization of an MSE can facilitate the occurrence of human rights abuses that are linked to structural human rights issues present in a certain host country or city. For example, if a host country has weak labour laws and limited protections in place, it is likely that the delivery of such an event will facilitate labour rights abuses due to the workforce that is usually needed to stage the event. The most prominent example here is obviously the Qatar World Cup, which has been criticized continuously since it has been awarded in 2010. The press and civil society regularly publish reports that document the exploitation of migrant workers on Qatar's (World Cup) construction sites, uncovering modern slavery-like conditions and the unsafe working conditions that some of them are facing, leading to injuries and death (9). Other risks related to structural human rights issues that hosts are facing come from increased security demands. When MSEs take place in “geopolitically unstable environment[s]”, there are higher risks of terrorist attacks for instance, which materialized in the case of the Sochi Winter Games, where just weeks before the event started a series of attacks occurred (10). With the amount of deployed security personnel rising, there is also an increased readiness to use violence. This is in particular a problem for those host cities, in which security structures are already fragile. As the 2016 Olympics in Rio approached, killings of in particular young black males living in favelas in the context of the police pacification campaigns increased (11). Amnesty International reported that in the months leading up to the event, violence and police killings increased every month (12). In South Africa, some regions showed an almost 50% increase in fatal shootings by the police in the two years leading up to the World Cup (13).

These examples show that MSEs can have adverse impacts on the entire range of human rights, directly, and indirectly. Examples of direct impacts range from violations of the freedom of expression and the right to information and participatory rights, to violations of privacy rights, housing rights, from child labour, to forced labour, and to arbitrary arrests and discrimination. Indirect impacts can occur as a consequence of these direct human rights abuses. For instance, when communities are forcefully evicted to make room for the infrastructure needed to stage the event, that is a human rights abuse directly related to the event. If as a consequence people lose their livelihoods, or access to education or participation in cultural or social life, that can be considered as human rights abuses indirectly linked to the delivery of an event. What these examples also show is that a wide range of rights-holders, including specific groups like children, women and girls, migrant workers, or persons with disabilities can potentially be impacted and that these abuses can happen at all stages of an event's lifecycle, from the bidding stage, to the preparation stage, at games' time, and even in the aftermath of the event (14). In particular when human rights harms that occurred before or during the event have not been remedied, these adverse human rights impacts continue even when the event has finished.

## The complexities of MSE governance

The problem with addressing these cases is that these events are highly complex operations, jointly staged by a mix of national, international, public, and private actors and organized based on a complex framework of contracts and agreements between all those actors. While International Sports Governing Bodies (ISGBs) like the Fédération Internationale de Football Association (FIFA) or the International Olympic Committee (IOC) formally own these events, also state actors in form of hosting authorities from different levels of government, from central to municipal, as well as (multinational) sponsors, agencies, broadcasters and companies are involved (15). All these actors are somehow linked to each other, by contracts and agreements. In the planning stage of the 2012 Games in London, around 2,200 contracts have been signed (16). This creates a complex web of frameworks and laws that apply.

This peculiar and pluralist legal framework is based on three different legal frameworks that overlap: *lex sportiva*, domestic laws, and human rights law. *Lex sportiva* refers to the regulation of international sports, based on contracts and private regulations issued by sports governing bodies like FIFA and the IOC, and the jurisprudence of the Court of Arbitration for Sport (17). Other rules and laws that event owners and organizers have to take into account when bringing an MSE to a certain host country are domestic labour laws, tort laws, immigration laws, intellectual property laws, competition or tax laws play a role for the various interactions and operations related to MSEs (18). In addition, the applicable domestic laws also include those laws that have been adopted specifically for the event, so-called Olympic Laws or World Cup Acts. The different actors involved also have to abide by the domestic laws and rules of their home country, including Swiss law applicable to the event owners, as FIFA and the IOC for instance are both registered in Switzerland as associations. Human rights law becomes applicable increasingly, primarily due to recent changes in the bidding and hosting regulations, and secondarily through statutory and policy commitments. In 2017, both the bidding and hosting regulations for the Olympic Games and the World Cup have been amended to integrate human rights standards and requirements for bidders and hosts.

In addition to this multi-jurisdictional nature of MSEs, another factor that increases the complexity of their governance and organization is that hosting these events often comes with a certain climate of exceptionalism, that also extends to the legal field. The different rules and laws that apply position the organization and delivery of MSEs in between public and private ordering, which adds to what Corrarino has called a climate of legal exceptionalism (19). It is caused by strict requirements and high expectations, as well as the tight time schedules, which lead to regular legal processes being side-lined and exceptional legal regimes to take over (20). In practice, decisions are being fast-tracked, for example by lowering standards of due process, circumventing normal public procurement procedures and loosening participatory rights of citizens or worker's safety standards (21). The worldwide enthusiasm for these events and the global spotlight they receive from media, the chances that national and transnational elites see in those events to push forward their political agenda further add to this exceptionalist culture around MSE (20). The consequence of this exceptionalism is not only an undermining of human rights but these exceptional legal regimes also tend to undermine options to hold the relevant actors accountable (22).

## Towards shared responsibility and collaborative remedy

Searching for ways to address MSE-related human rights abuses means to identify those responsible and hold them accountable. However, this comes with considerable challenges. Due to the plurality and diversity of actors involved, you face the problem of many hands, as it has often been referred to (23). It describes “the phenomenon that, due to the complexity of the situation and the number of actors involved, it is impossible or at least very difficult to hold someone reasonably responsible” (24). As a result, the lines of responsibility and accountability for actions taken in the context of staging MSEs, but also for harms that happen linked to these events, are blurred, which makes it difficult for rights-holders to understand what happened, identify the responsible parties, and hold them to account. Moreover, it can facilitate blame-shifting between the different actors and even lead to a number of actors escaping their responsibility entirely. Furthermore, due to the multi-jurisdictional nature of these events, different laws and standards apply to the different actors involved. So even if it is possible to identify responsible parties, it is very unlikely that they are bound by the same standards, which would mean that each of them would have to be held accountable separately under the relevant norms and systems.

A potential way forward is adopting a shared responsibility approach to the organization of these events, as has been researched and argued extensively in a recent study (25). Like the name suggests, the idea of shared responsibility is to share responsibility between the different involved actors (26). The more general meaning is very present in the MSE and wider sporting context, in multiple ways. For instance, when FIFA launched an oversight body to monitor the systems in place to ensure decent working conditions at FIFA World Cup construction sites in Qatar, it stated that “labour issues especially in the construction sector are a global challenge and we understand that everybody involved has a shared responsibility” (27). Likewise, a study from the MSE Platform for Human Rights in 2016 stated that “MSEs are a shared responsibility and demand shared responses” (28). Furthermore, academic publications on the topic speak of “teamwork” to address adverse human rights impacts of MSEs (3).

However, in practice this idea seems barely implemented when it comes to human rights impacts of these events. Generally, MSE organizers and sports bodies usually have a quite clear understanding of the different tasks and responsibilities related to staging an MSE, some even had a responsibility matrix in place (29). But this did not yet include responsibilities for respecting and protecting human rights. To fully embrace a shared responsibility approach to human rights impacts of MSEs would mean to on the one hand apply it from the outset, so in a preventative and forward-looking function, which would map human rights risks, clarify responsibilities in terms of addressing and mitigating those risks, thereby make the organization of these events more transparent, and help those affected understand who is responsible for what. On the other hand, it needs to apply in a retrospective way, to ensure that when harm has happened, there are ways to hold all actors that contributed in one way or another to account based on their share of contribution, to ensure that those affected have access to remedy.

An integral part of this shared responsibility approach should be collaborative remedy. As such, access to remedy is another challenge on its own in the sporting context, for many reasons, one of them being that most available mechanisms in the sporting context have not been designed with human rights harms in mind and therefore lack expertise and capacity (30). In the MSE context, the challenge is that relevant mechanisms usually have a limited scope and cover only specific types of actors involved, and there is no central mechanism that can hold all actors to account. This provides significant obstacles for rights-holders. If following this idea of shared responsibility, a natural next step would be to also consider collaborative remedy, in the absence of a central mechanism. What collaborative remedy could mean in practice, is that different mechanisms relevant in the context of MSE-related human rights abuses find a formal way to collaborate in cases that raise questions of shared responsibility, to avoid blame shifting and that some actors escape responsibility, and to ensure that rights-holders get compensated for the entire harm they suffered and not just parts of it if any at all. What that could also mean, is that relevant actors collaborate for *ad hoc* remedy initiatives. The recent call for a comprehensive programme to remedy all labour abuses to which FIFA contributed in the context of the Qatar World Cup is a good example for that. In a joint letter, Amnesty International and Human Rights Watch, together with other civil society organizations, urge FIFA, the Qatari government, trade unions and the ILO to work together to establish a remedy system and ask FIFA to reserve “an amount not less than the US$440 million prize money offered to teams participating in the World Cup, to be invested in funds to support remediation” (31).

While these concepts are interesting ideas in theory, what matters is the changes we see in practice. In fact, with an evolving sport and human rights movement that is reaching a number of stakeholders in sport's ecosystem, we do see changing practice. Even though this movement goes beyond MSEs, it is safe to say that the increased attention to human rights impacts of MSEs from the past decades, and also the historical link of MSEs with human rights causes and protests and other social movements, helped this movement to gather speed. A limited selection of important moments in that journey include campaigns by Human Rights Watch and other NGOs around the Beijing Olympics bid in 2000 and the actual event in 2008 (32), the awarding of the 2018 FIFA World Cup to Russia and the 2022 FIFA World Cup to Qatar in 2010, and the open letter sent by John Ruggie, author of the United Nations Guiding Principles for Business and Human Rights (UNGPs), and Mary Robinson, former UN High Commissioner for Human Rights, to then FIFA President Blatter urging FIFA to fully integrate human rights considerations into its decision making in 2014 (33). The latter certainly helped to bring FIFA on the human rights journey and sparked broader discussions on the human rights issues in the world of sport more generally (3). For these discussions, the UNGPs provide the authoritative framework, according to which a sport body has a responsibility to respect human rights, which increasingly finds acceptance in the sporting world.

Currently, we see traces of that movement in different areas of the sport's ecosystem, from measures taken by different international sports bodies, such as including human rights provisions in event bidding and other-sport related regulations, and human rights strategies and policies being adopted. In addition, there are developments on the national level. The German football association has adopted a human rights policy (34), and many national teams made statements in relation to the situation in Qatar. There are discussions and negotiations held by international and regional intergovernmental organizations to adopt new sport policies that include reference to human rights, and there are national initiatives around the world for setting up safe sport entities (35) or establishing athlete representative bodies, and we see continuing and more pressure from civil society, with more trade unions or athlete unions integrating the topic, human rights organizations conducting studies into not only MSEs but also human rights issues linked to day-to-day sports, and organizations like the Centre for Sport and Human Rights emerging and growing.

## Making MSEs & human rights sustainable

In order to make the aforementioned developments lasting and the increase in knowledge and capacity sustainable to ensure that future generations working in the world of sport or related fields can consolidate these recent developments, research and education are key. In fact, the recent changes were accompanied by a growing body of literature on human rights and sport, and human rights and MSEs more specifically (36). Since 2018, the project “EventRights” conducts research on how the bidding, planning and delivery processes for MSEs can more effectively protect and promote the rights of affected groups and how research methods to help better understand the impact of MSEs on human rights can be developed and utilised to create the conditions for change (37). As part of the outcomes of this project, an article was published in which a conceptual model is proposed for how progressive human rights outcomes can be achieved in the context of bidding, planning and implementing MSEs, and an online event organized on “Foregrounding a Rights-Based Agenda for Sport Events”, which triggered the Resesarch Topic in which this piece is published (38). In 2019, the book “Dark Sides of Sport” was published, which combines contributions of leading international scholars on historical and contemporary challenges for sport, including that of human rights and MSEs (39). In 2019, the Tilburg Law Review published a special issue on FIFA and human rights, which is the result of an academic conference on the topic held in May 2019 (40). In the domain of legal research in particular, the International Sports Law Journal published a number of articles in the past years that deal with human rights issues of MSEs and day-to-day sport, and as most recent publication a series of papers on remedy and redress for sport-related human rights abuses. At the moment of writing, a number of book projects on the topics of sport and human rights, and MSEs and human rights, are in the making.

However, there is a lot of room and also need for more research into the different areas that are linked to sport and human rights, to have an evidence base that helps to call for change and improvement. Much of the work that is out there, studies, articles, books, rely heavily on studies by NGOs like Human Rights Watch, Transparency International, Amnesty International, UNICEF, and others. This can and should be supported by more scientific research in those areas where there is a lack of research. For example, we know less on human rights impacts of single sport events, smaller sport events, or e-sport events, there is no comprehensive research on how women and girls are impacted differently by MSEs, and we also know less about how MSEs can best be used to promote human rights. Regarding sports more generally, there is for example no comprehensive database on safeguarding policies and procedures and how effective they are, or on available remedy mechanisms for sport-related human rights abuses. Sports bodies, in particular the leading international ones as owners of MSEs and rule-makers of the respective sport are well positioned to fund more research in this field, while independence of the research must be guaranteed.

In terms of education, there is an underused opportunity to teach sport and human rights at primary, secondary, tertiary and professional levels. Using sport to teach human rights values can and does already start in primary schools, were physical exercise is often an integral part of the education, where human rights values in form of fair play, equality, non-discrimination can be taught and learned in a playful manner. This can be continued and expanded in secondary education, where additional emphasis could be put on the broader human rights risks related to sport, in form of what is expected behaviour, what constitutes harm, how can it be prevented and addressed, and how sport is performed and perceived around the world and in different cultures, including in the MSE context. University studies and other educational programs after school provide a lot of room for integrating in-depth sport and human rights education, both in human rights related study programs, where there is currently hardly any mention of sport as a risk to human rights but also no mention of sport as opportunity to advance human rights. The same is true the other way around for the variety of sport-related programs, sport management, sport history, sport policy, sport sociology, sport law, and others. Currently, human rights studies usually do not deal with sports and sport studies barely touch upon human rights.

With the evolving sport and human rights movement, this seems to be changing gradually. A number of sport ethics and integrity or sport law programs have started to include human rights, as a sub-topic, for instance with lectures specifically dedicated to sport and human rights, but also entire courses or workshops on sport and human rights are emerging. The same is true for professional education, as more workshops, webinars and entire courses on topics related to sport and human rights are being offered. This rise in offerings confirms the existence of a sport and human rights movement, and that the issue of mega-sporting events and human rights has grown into wider considerations of the human rights risks, but also opportunities, linked to sports more generally. Strengthening education and research in this field, and securing impact for these initiatives, will make the movement sustainable and the evolving good practice long-lasting, so that negative human rights impacts of MSEs and sports can be further minimized and the potential to promote human rights better realized.
